# Fluoride Adsorption from Aqueous Solution by Modified Zeolite—Kinetic and Isotherm Studies

**DOI:** 10.3390/molecules28104076

**Published:** 2023-05-13

**Authors:** Thouraya Turki, Abdelkader Hamdouni, Alexandru Enesca

**Affiliations:** 1 Natural Water Treatment Laboratory, Water Researches and Technologies Center (CERTE), Technopark of Borj-Cedria, P.O. Box 273, Soliman 8020, Tunisia; 2 High Institute of Sciences and Technology of Environment of Borj Cedria, University of Carthage, Carthage 1054, Tunisia; 3 Product Design, Mechatronics and Environment Department, Transilvania University of Brasov, Eroilor 29 Street, 500036 Brasov, Romania

**Keywords:** fluoride, modified zeolite, adsorption isotherm, kinetics models

## Abstract

Fluorine is a very common element in the Earth’s crust and is present in the air, food, and in natural waters. It never meets in the free state in nature due to its high reactivity, and it comes in the form of fluorides. Depending on the concentration of fluorine absorbed, it may be beneficial or harmful to human health. Similar to any trace element, fluoride ion is beneficial for the human body at low levels, but as soon as its concentration becomes too high, it is toxic, inducing dental and bone fluorosis. The lowering of fluoride concentrations that exceed the recommended standards in drinking water is practiced in various ways around the world. The adsorption process has been classified as one of the most efficient methods for the removal of fluoride from water as it is environmentally friendly, easy to operate, and cost-effective. The present study deals with fluoride ion adsorption on modified zeolite. There are several influential parameters, such as zeolite particle size, stirring rate, solution pH, initial concentration of fluoride, contact time, and solution temperature. The maximum removal efficiency of the modified zeolite adsorbent was 94% at 5 mg/L fluoride initial concentration, pH 6.3, and 0.5 g modified zeolite mass. The adsorption rate increases accordingly with increases in the stirring rate and pH value and decreases when the initial fluoride concentration is increased. The evaluation was enhanced by the study of adsorption isotherms using the Langmuir and Freundlich models. The Langmuir isotherm corresponds with the experimental results of the fluoride ions adsorption with a correlation value of 0.994. The kinetic analysis results of the fluoride ions adsorption on modified zeolite allowed us to demonstrate that the process primarily follows a pseudo-second-order and then, in the next step, follows a pseudo-first-order model. Thermodynamic parameters were calculated, and the ΔG° value is found to be in the range of −0.266 kJ/mol up to 1.613 kJ/mol amidst an increase in temperature from 298.2 to 331.7 K. The negative values of the free enthalpy ΔG° mean that the adsorption of fluoride ions on the modified zeolite is spontaneous, and the positive value of the enthalpy ∆H° shows that the adsorption process is endothermic. The ∆S° values of entropy indicate the fluoride adsorption randomness characteristics at the zeolite-solution interface.

## 1. Introduction

Water is a vital resource for all living creatures. It covers three-quarters (3/4) of the Earth’s surface and represents about two-thirds (2/3) of the human body [1]. Sources of fresh water are manifold, but surface drinking water and groundwater are the commonest. Indeed, most countries in the world rely on both sources to secure public water supplies. The issue that arises in countries such as Morocco, Algeria, Senegal, and Tunisia is the existence of excessive amounts of fluoride in drinking water [2]. In the South of Tunisia, and in the Gafsa region in particular (Metlaoui, Omlarayes, and Redayef), the water contains high fluoride concentrations—approximately 2 to 4 mg/L, which exceeds the WHO’s target (1.5 mg/L). Fluorine is an essential and beneficial chemical element for the human body if ingested at low rates. However, dangerously high levels of fluoride in water supplies can lead to serious health problems. At 0.5 mg/L of fluoride ions, water plays a prophylactic role in preventing cavities and early tooth decay through enamel remineralization. On the contrary, at higher concentrations (>0.8 mg/L), the risk of dental and skeletal fluorosis increases [3,4,5,6]. Bone fluorosis results from the excessive accumulation of fluoride in bones, causing changes in the skeleton structure and making them extremely fragile. In addition, an increased bone density (ribs, pelvic bone), ligament calcification, anarchic bone trabeculations structure, and a decrease in figurative element production in blood can be noticed [7].

In order to avoid public health problems linked to the consumption of drinking water rich in fluoride, the methods used to deal with excessive fluoride concentrations should be considered before the water is released to consumers. Conventional treatment processes (precipitation, adsorption, etc.) and unconventional (membrane separation processes) have been implemented to reduce excess fluorine to concentrations that meet the recommendations of the World Health Organization (WHO) and the standards in place in the concerned countries. Our literature study indicated that adsorption appeared to be the most appropriate method for fluoride removal in terms of material consumption and treatment costs (energy consumption). To a lesser extent, adsorption on modified zeolite could be utilized due to its high rate of fluoride ion removal [8,9]. Studies and applications of zeolites have increased considerably in recent decades, and a large number of publications and patents have been issued [10,11,12,13]. Natural zeolites have been increasingly substituted by synthetic zeolites, and today, more than 150 different crystal structures of the material are known [14]. Although very little is used in industrial processes [15], thousands of tons of zeolites are consumed each year to remove hardness from water as catalysts, adsorbents to control soil pH, and as a fertilizer to adsorb moisture [8]. Low-cost metal-laden adsorbents have attracted a lot of attention from researchers in this field, as they have greatly improved selectivity and adsorption capacity [16,17].

This study focuses on finding sustainable solutions to reduce fluoride levels in Tunisian drinking water by employing a modified type of zeolite as an adsorbent material. Clinoptilolite was modified to increase its affinity for fluoride ions, and the adsorption processes were evaluated. Clinoptilolite has been used for the removal of lead [18], tetracycline [19], ammonium [20], and fluoride [17] from wastewater. Compared with other similar works [21,22], this paper indicates the steps required to establish the optimized dosage of modified clinoptilolite in order to acquire the maximum fluoride absorption capacity. Additionally, the influence of various parameters such as pH, contact time, temperature, and initial fluoride concentration was analyzed. The isothermal behavior and adsorption kinetics for fluoride adsorption onto the modified zeolite were investigated, and the fluoride adsorption mechanism was evaluated.

## 2. Results and Discussion

### 2.1. Material Characterization

**Figure 1:**
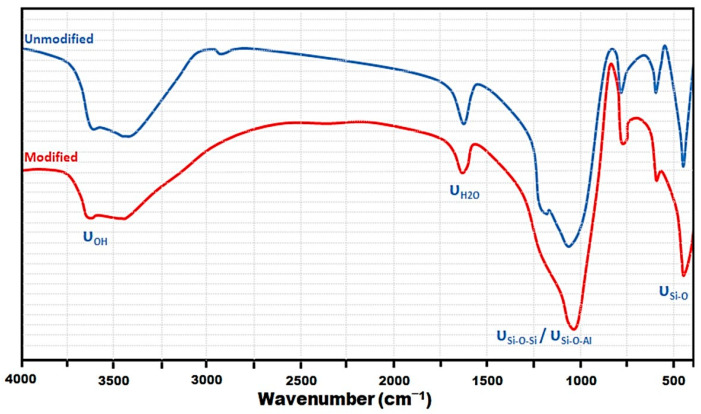
FTIR spectra of the activated zeolite.

FTIR spectra were performed using a VERTEX 70 Fourier transform spectrometer over a range from 400 to 4000 cm^−1^ with a resolution of 4 cm^−1^. The modified zeolite was packaged as dispersion in a KBr pellet. The IR spectrum of the modified and unmodified zeolite is shown in Figure 1. As expected, there are no significant changes in the FTIR spectra due to the treatments, and the main bands are present in both materials. The exchange in the band intensity is a consequence of the diffusion processes induced during the thermal treatments [23].

The broad band between 3500 and 3700 cm^−1^ in the activated zeolite shows the presence of two species on the adsorbent surface: free and hydrogen-bonded OH groups. In the OH stretching region, the infrared spectra show that there are hydroxyl groups attached and that zeolite is significantly hydrated. The bands centered at 3444 cm^−1^ and 1600 cm^–1^ refer to water molecules associated with Na and Ca in the adsorbent channels and cage’s structure. The 1065 cm^−1^ band indicates the asymmetric stretching vibration modes corresponding to the internal Si-O-Si or Si-O-Al bonds. The 800 cm^−1^ and 550 cm^–1^ bands are ascribed to the Si-O-Si or Si-O-Al bonds groups stretching vibration modes, as well as the Si–O bonds bending vibrations. These results are in agreement with other articles [24], showing that the main band corresponds to this type of modified zeolite.

**Figure 2:**
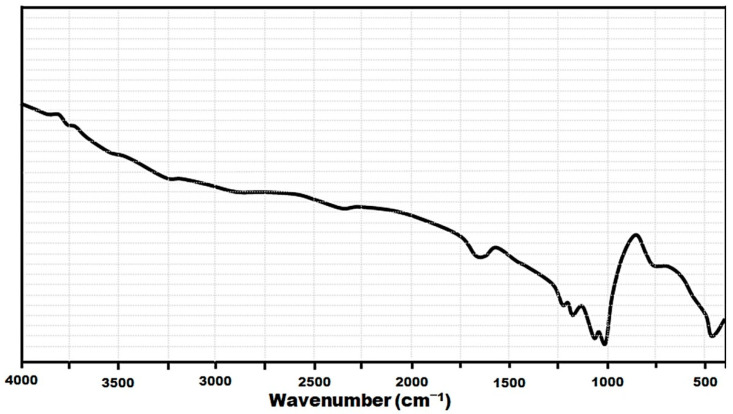
FTIR spectra of the zeolite after the adsorption of fluoride ions.

After adsorption experiments (Figure 2) were conducted, slight shifts and amplitude modifications corresponding to some bands were observed, indicating an interaction between OH groups or active metal (aluminum, silicon, and iron) surfaces with fluoride ions [25,26]. There is also a decrease in band intensity attributed to the group at 3570 cm^−1^ after the adsorption process, which is associated with the exchange between the fluoride and hydroxide radicals.

**Figure 3:**
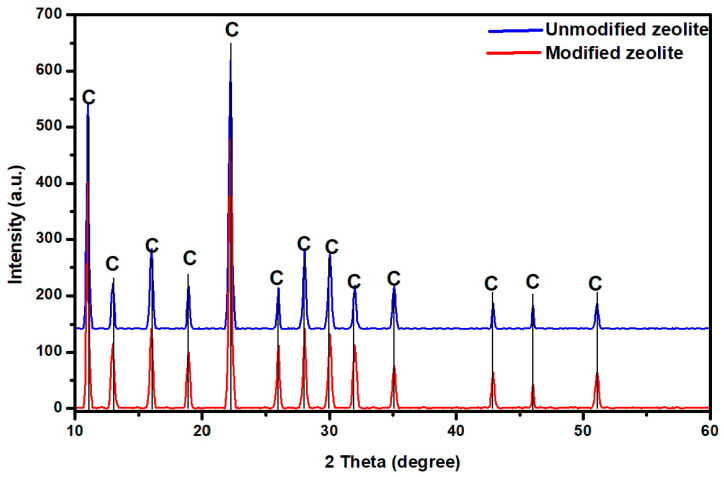
Diffraction patent corresponding to unmodified and modified zeolite.

The diffraction analysis presented in Figure 3 indicates the presence of characteristic lines corresponding to clinoptilolite (JCPDS-ICCD 98-000-2606) with a predominant monoclinic structure. The diffraction patent of unmodified and modified zeolite exhibits the same diffraction line, with the only difference being that, for the modified zeolite, the minor lines have a higher intensity due to the thermal treatment of the adsorber [27].

**Figure 4:**
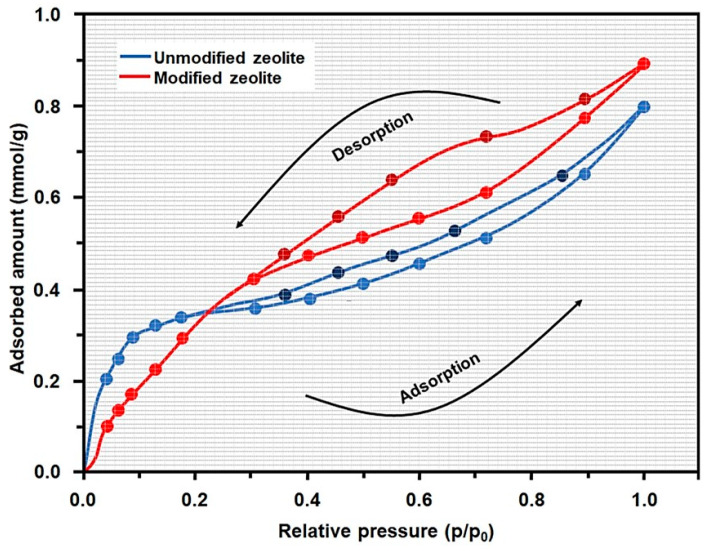
Nitrogen adsorption/desorption isotherms corresponding to unmodified and modified zeolite.

The nitrogen low-temperature adsorption/desorption isotherm (Figure 4) corresponds to a IV-type isotherm with H3-type loop hysteresis in accordance with IUPAC classification [28]. Both materials present a relatively low specific surface area of 24.4 m^2^/g for unmodified zeolite and 29.7 m^2^/g for modified zeolite. The increase in specific surface area corresponding to the modified zeolite can be associated with the presence of a larger number of free micropores and not necessarily the formation of new micropores.

### 2.2. Influence of the Adsorbent Amount

During this study, the modified zeolite with two morphologies (powder and larger granular (≤45 µm)) was tested, and the adsorbent quantity was varied from 0.1 to 0.6 g while fixing the fluoride concentration.

**Figure 5:**
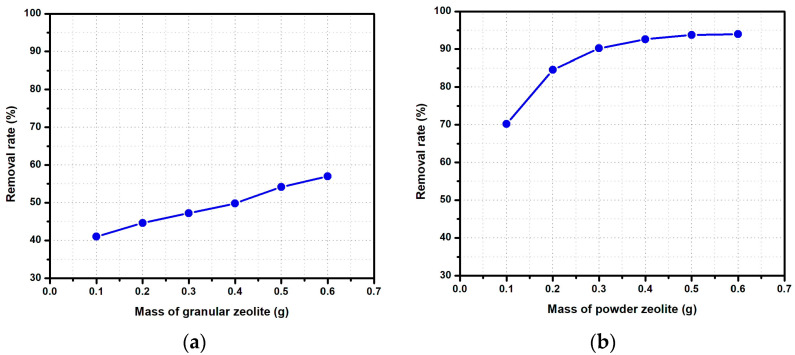
Variations in the fluoride ion removal rate function of adsorbent dosage: (**a**) granular modified zeolite and (**b**) powder-modified zeolite ([F^−^] = 5 mg/L, pH = 6.3, T = 30 °C).

The experiments indicate a relative linear dependency (Figure 5a) between the fluoride removal rate and the amount of large granular modified zeolite. The adsorption increases from 41% for 0.1 g of zeolite to 57% for 0.6 g of zeolite added in the working solution after one hour of contact, at room temperature and without stirring. However, when powder-modified zeolite is added, there is a significant increase in the fluoride removal rate (Figure 5b), especially when the adsorbent quantity is increased to 0.2 and 0.3 g. It must be underlined that using 0.3 g of powder-modified zeolite is enough to remove >90% of fluoride ions. After 0.3 g zeolite dosage, there is a slow increase in the fluoride removal rate due to the fact that the available fluoride ion concentration is reduced. These results are in agreement with the work of Papari et al. [29], indicating that the removal efficiency of fluoride (10 ppm solution concentration) increased from 15.86% to 82.24% when the adsorbent dose of powdered Conocarpus biochar increased from 2 to 15 g/L. Consequently, all the other experiments were performed using powder-modified zeolite.

### 2.3. Influence of the Stirring on Fluoride Adsorption (Dynamic vs. Static)

**Figure 6:**
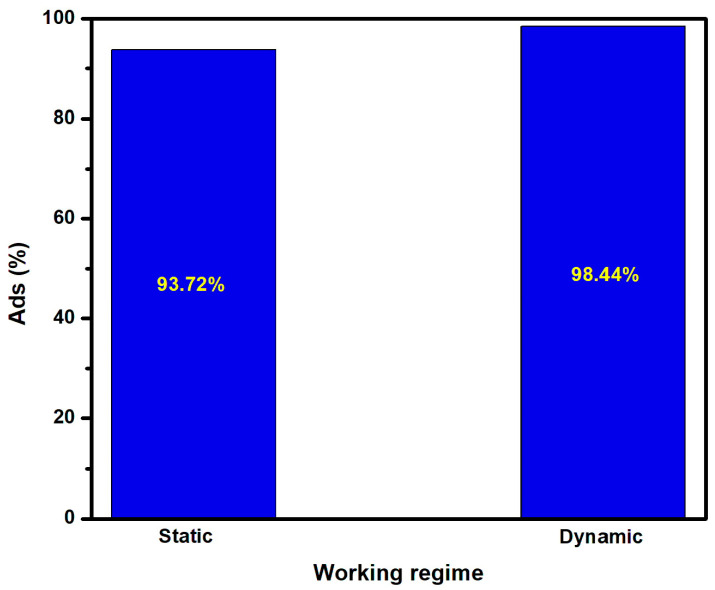
Fluoride ion adsorption in static and dynamic working regimes.

The influence of stirring vs. static experiments on the removal of fluoride ions was evaluated, and the results are presented in Figure 6. Both experiments were performed with the same quantity of adsorbent optimized in Section 3.2, and the stirring speed was 350 rpm. The results indicate that working in a dynamic regime will increase the fluoride ion adsorption rate from 93.7% to 98.5%.

The higher removal rate in the dynamic working regime suggests that the diffusion can be tailored by using the stirring process to decrease the liquid film captive on the adsorbent interface. This experimental observation corresponds with the work described by Pricila et al. [30], which showed that the adsorption will be favored when stirring ensures a uniform distribution of fluoride ions at the liquid–solid interface.

### 2.4. Influence of Fluoride Initial Concentration and Contact Time

**Figure 7:**
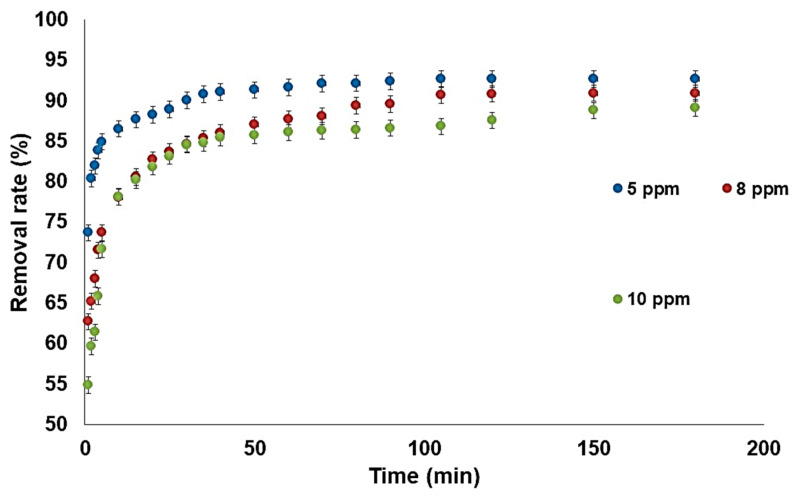
Effect of the initial fluoride concentration (m_adsorbent_ = 0.5 g, pH = 6.3, T = 28 °C, V = 100 mL).

The influence of the initial fluoride concentration on the adsorption rate was evaluated, and the results are presented in Figure 7. Three solutions with 5 ppm, 8 ppm, and 10 ppm initial fluoride concentrations were used. The curve shape highlights two main zones:

1. Between 5 min and 50 min, the removal rate is faster due to the higher number of available active sites on the adsorbent surface.
2. Between 50 and 180 min there is the second zone, which is similar but with a plateau, where the removal rate decreases due to the synergic action, the lower number of available active sites, and fewer available fluoride ions; this behavior was also observed by Tan et al. [31] and was described as a phase of pseudo-equilibrium between the rates of adsorption and desorption.

The kinetic study indicates that a contact time of 90 min is generally sufficient to reach a state of equilibrium. Upon comparing the curves corresponding to each concentration, it is evident that the fluoride adsorption rate increases when the initial concentration of fluoride ions is higher. This decrease in the rate of elimination is explained by the presence of excess fluoride ions in the solution, which hinders adsorption [32,33,34].

**Figure 8:**
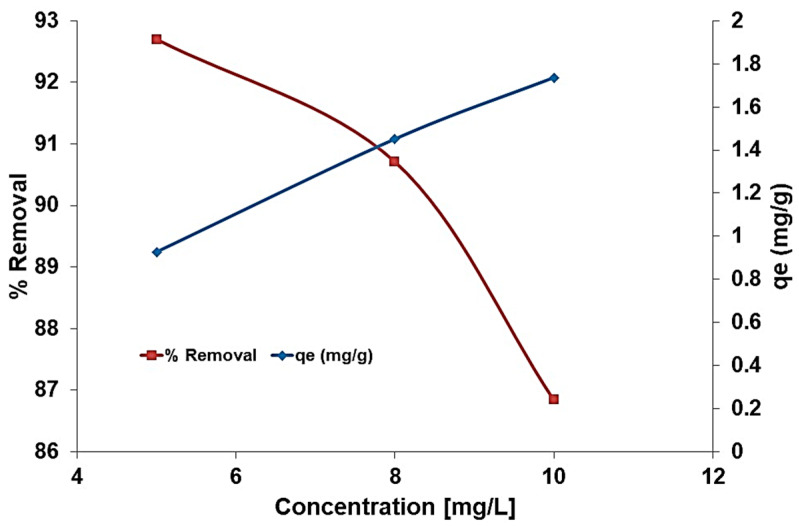
Effect of initial fluoride concentration on adsorption capacity and removal rate.

A reduction in removal percentage at higher fluoride concentrations may be a consequence of the fluoride ions competing for the available adsorbent binding sites. As presented in Figure 8, the adsorption capacity increased from 0.93 to 1.74 mg/g as the initial fluoride ion concentration increased from 5 to 10 mg/L, which is in agreement with Kebede et al. [35], who found that the activity of fluoride ions increases as their concentration increases. Table 1 presents a comparison between the adsorption capacity of different adsorbers used for the removal of fluoride from wastewater. As observed, higher adsorption capacities can be reached by increasing the adsorbent dosage. However, at an adsorbent dosage of 0.5 g/L, the modified clinoptilolite exhibits higher or similar absorption capacities (1.74 mg/g) compared to aluminum-modified zeolite (0.47 mg/g), marble apatite-CM (0.25 mg/g), or lanthanum oxyhydroxides anchored commercial granular activated carbon (1.79 mg/g).

### 2.5. Influence of pH on the Fluoride Ions Removal

**Figure 9:**
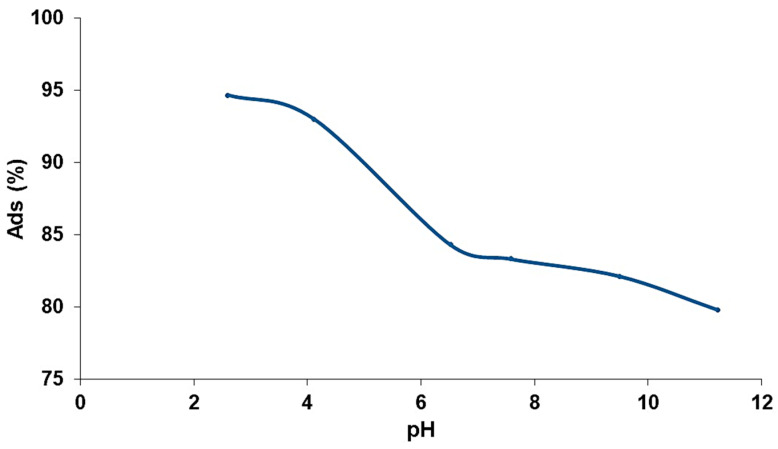
Influence of pH on fluoride ion adsorption.

**Figure 10:**
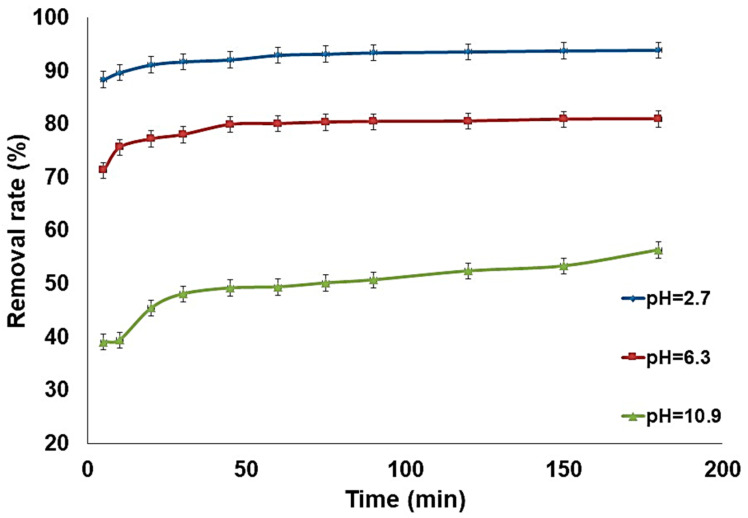
Evolution of removal efficiency at different pH (m_adsorbent_ = 0.5 g, [F^−^] = 5 mg/L, pH = 6.3, T = 22 °C).

The pH is considered an important parameter in adsorption-based processes. In order to evaluate the influence of pH on the removal of fluoride ions, a series of tests at different pH were carried out. The pH was adjusted with solutions of hydrochloric acid (0.01 M) and sodium hydroxide (1 M), and the results are presented in Figure 9. The evaluation shows an interesting adsorption capacity, which reaches its maximum at 94% in an acid environment and decreases in an alkaline environment without exceeding 57%. The adsorption capacity is higher in acid pH and significantly decreases in alkaline pH. The adsorption in an acidic medium is enhanced due to the ability of fluoride ions to fix protons required for hydrofluoric acid. The reaction is strongly exothermic and considers the competition between the fluoride ion adsorption and complexation. The results are in agreement with Parashar et al. [41] who showed that the adsorption of fluorides increases at acidic pH, becomes constant up to pH = 8, and decreases in a basic medium. Additionally, the kinetic evaluation presented in Figure 10 confirms the superior adsorption capacity of the modified zeolite in acid pH. Adsorption in alkaline pH is lower because it is hampered by the presence of OH^−^ ions in the medium [42].

**Figure 11:**
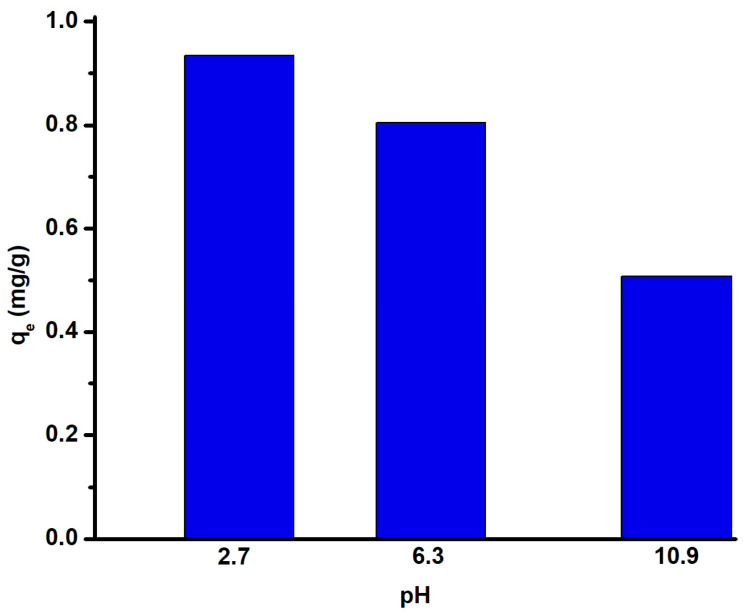
Adsorption capacity of modified zeolite (adsorbent) for fluoride at different pH.

As presented in Figure 11, the fluoride uptake capacity is not strongly affected when the pH is less than or equal to 7. To avoid affecting environmental factors, the results suggested that a pH value of 6.3 was the optimal pH for the removal of fluoride by the modified zeolite adsorbent. Even at a lower pH, increasing the adsorption capacity can be detrimental for large scale applications.

**Figure 12:**
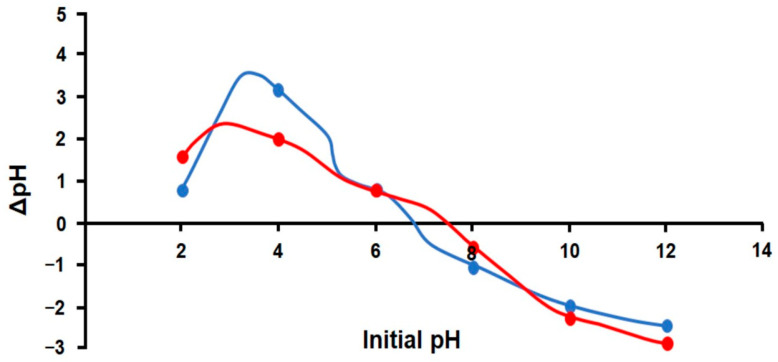
Determination of pH_pzc_ for unmodified (blue line) and modified (red line) zeolite.

The evaluation of pH_pzc_ (Figure 12) indicates that the modification made to zeolite has influenced the point of zero charge. The unmodified zeolite exhibits a pH_pzc_ value of around 6.8, while the modified zeolite pH_pzc_ was 7.6. Consequently, at pH values below 7.6, the modified zeolite surface will have a net positive charge, and at pH > 7.6, the surface will be negatively charged.

### 2.6. Influence of Temperature on the Fluoride Ions Removal

**Figure 13:**
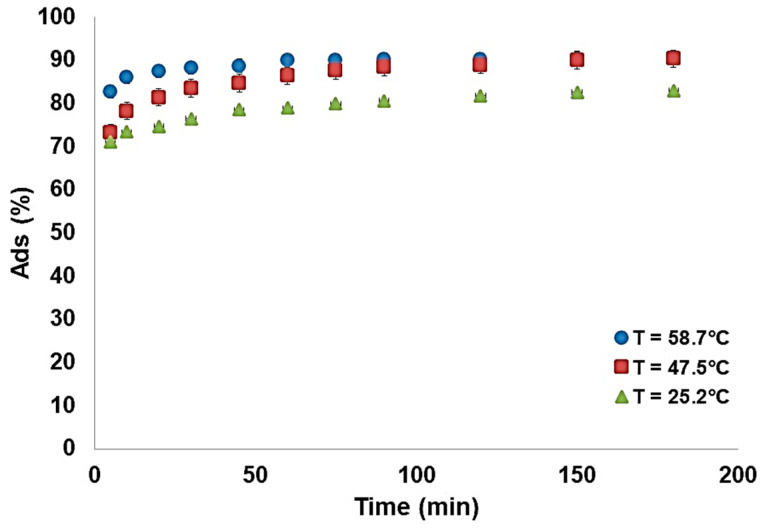
Adsorption of fluoride ions as a function of temperature (m_adsorbent_ = 0.5 g, [F^−^] = 5 mg/L, pH = 6.3).

Along with the pH, temperature is a major variable in the adsorption process that plays an important role in establishing the optimal working conditions for the modified zeolite. Our evaluation was performed at three different temperatures (25.2 °C, 47.5 °C, and 58.7 °C), and the results are presented in Figure 13.

**Figure 14:**
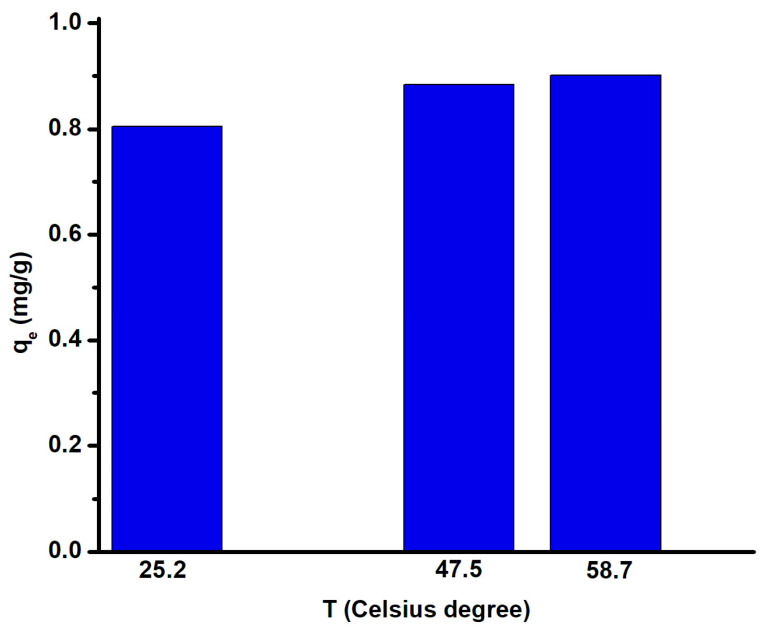
Influence of temperature on fluoride adsorption capacity.

The results indicate that the adsorption process is endothermic, meaning that the fluoride ion adsorption increases when temperature increases. This is a consequence of the fact that adsorption is controlled by diffusion, and diffusion increases when the temperature rises. This thermal correlation was also reported in other works [43,44,45] and was correlated with ion diffusion dependency on temperature. Figure 14 indicates that the highest adsorption capacity (0.9 mg/g) was obtained when the working temperature was 58.7 °C. The increase in temperature will also increase the rate of the adsorption process when equilibrium is reached via a chemisorption reaction [46]. Further increases in temperature are not recommended, as the evaporation process will reduce the overall solution volume and modify the fluoride concentration.

### 2.7. Influence of Co-Existing Ions on the Fluoride Ions Adsorption

**Figure 15:**
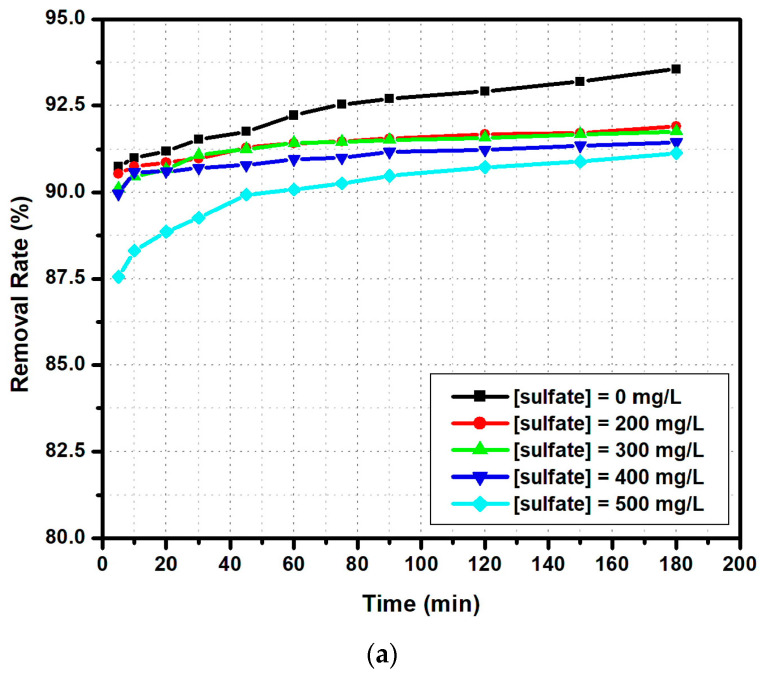

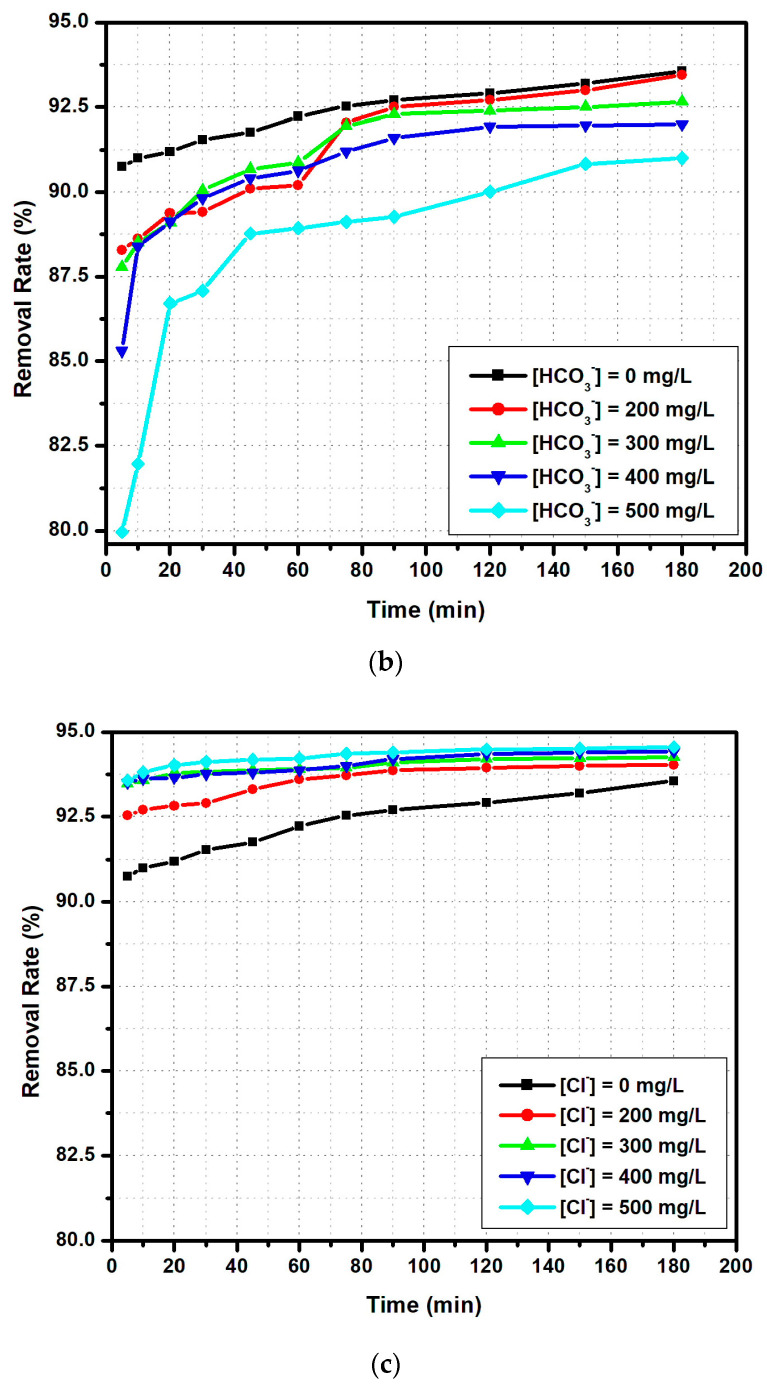
The influence of co-existing ions on fluoride anion adsorption (m_adsorbent_ = 0.5 g, [F^−^] = 5 mg/L, pH = 6.3, T = 30 °C, V = 100 mL): (**a**) sulfate, (**b**) hydrogen carbonate and (**c**) chloride ions.

In previous experiments, the synthetic solutions contained only fluoride ions. The study of other anion interferences, in particular sulfate, hydrogen carbonate, and chloride anions, on the fluoride ion adsorption capacity by the modified zeolite is illustrated in Figure 15.

In the presence of sulfate anions (Figure 15a), the highest influence was observed at 500 ppm, when the final percentage of adsorbed fluoride ions decreased to 90%, compared to 93% when no sulfate was added. It is important to underline that the main differences between the lower and higher sulfate concentrations were observed in the first 50 min of the experiment, indicating the competition among ions for the available active sites from the modified zeolite surface. A similar result was described in another paper [22], which showed that, at concentrations greater than 300 ppm in sulfate, chloride, and hydrogen carbonate, the adsorption of fluoride ions decreased [47]. This tendency seems to be more obvious at higher concentrations (500 ppm). A similar behavior was observed when a hydrogen carbonate anion was added (Figure 15b). Indeed, by increasing the concentration of hydrogen carbonate ions, the adsorption percentage decreases. These results can be explained by the fact that these ions replace fluoride ions at the adsorption sites. The work done by Zhijie et al. [48] indicates that, depending on the adsorbed substrate, a hydrogen carbonate ion concentration higher than 200 ppm will decrease the fluoride ion removal rate. The presence of chloride anions seems to enhance the fluoride ion removal rate (Figure 15c), as both are halide anions. According to Drouichea et al. [22], chloride anions act as a conditioning substrate factor on zeolites and other alumina-silicate materials, inducing the formation of more active sites ready for fluoride anion adsorption.

### 2.8. Kinetics of Adsorption

The pseudo-first-order model is deduced from the following differential equation (Equation (1)) [24]:

(1) $$\frac{dq_{t}}{dt}=k_{1}\cdot \left(q_{e}-q_{t}\right),$$

which is integrated into the boundary conditions from (*t* = 0, *q* = 0) to (*t*, *q*) and linearized to obtain the next equation (Equation (2)):

(2) $$\log\left(q_{e}-q_{t}\right)=\log\left(q_{e}\right)-\frac{k_{1}}{2.303}t,$$

where *k*_1_ is the first order rate constant (min^−1^), *t* represents the contact time (min), *q_e_* express the amount of fluorine adsorbed per unit mass at equilibrium (mg/g), and *q_t_* is the amount of fluorine adsorbed per unit mass of adsorbent at time *t* in mg/g. The values of *k*_1_ and *q_e_* can be calculated by plotting log(*q_e_* − *q_t_*) as a function of *t*.

**Figure 16:**
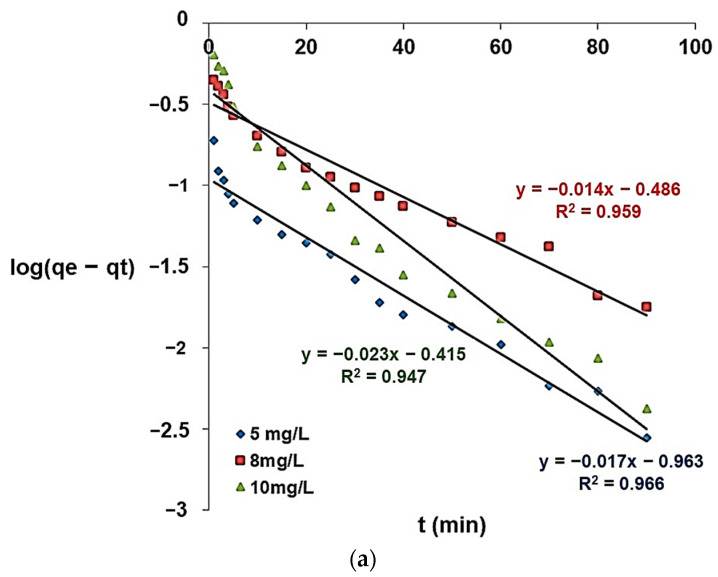

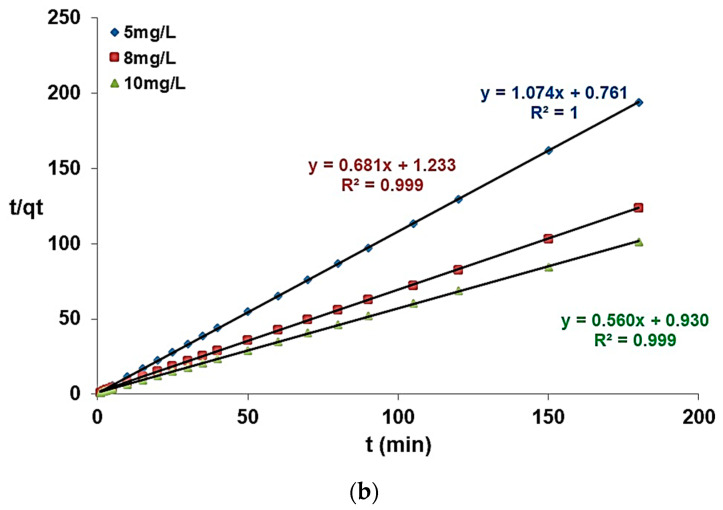
Pseudo-first-order kinetic model (**a**) and pseudo-second-order kinetic model (**b**) for fluoride adsorption.

The pseudo-first-order and pseudo-second-order kinetic models for the adsorption of fluoride ions are given in Figure 16, along with Table 2, which contains the kinetic parameters.

The pseudo-second-order model corresponding to the Heckscher–Ohlin model, also known as the pseudo-second-order model [24], considers the type of adsorption to be chemisorption. The mathematical model is expressed using the following equation (Equation (3)):

(3) $$\frac{dq_{t}}{dt}=k_{2}{\left(q_{e}-q_{t}\right)}^{2},$$

Considering the process rate and after linearization, the equation takes the following expression (Equation (4)):

(4) $$\frac{t}{q_{t}}=\frac{1}{k_{2}\cdot q_{e}^{2}}+\frac{1}{q_{e}}t,$$

According to Table 2, the correlation coefficient closer to 1 corresponds to the pseudo-second-order model. Therefore, this model can be used to describe the experimental results for the fluoride ion adsorption on the modified zeolite. The pseudo-second-order model was also suitable to describe fluoride adsorption onto brushite [49], alumina of an alkoxide nature [50], and charcoals [51].

The transfer of a fluoride from an aqueous phase to a solid phase follows four steps, which can be either independent of each other or simultaneous. The first is represented by the transfer of the fluoride from the aqueous phase to the surface of the solid. The second step represents the interparticle spaces diffusion (also known as external diffusion). The next step is associated with intraparticle diffusion, and finally, the last step considers the surface chemical reactions. It should be noted that the first step can be tailored in the dynamic processes, while the last one is rather fast, suggesting that the diffusion processes most likely limit the adsorption step.

**Figure 17:**
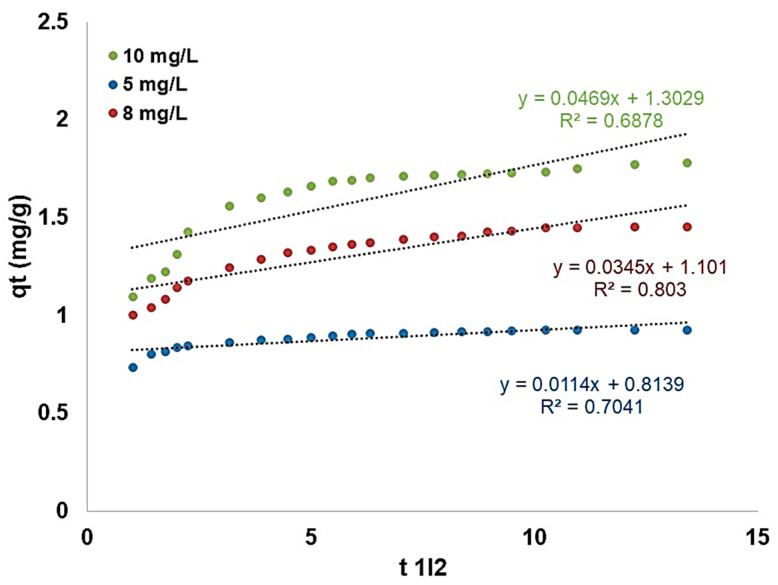
Representation of intraparticle diffusion of fluoride adsorption on modified zeolite.

In order to identify the diffusion mechanism, the kinetic results were then analyzed using the intraparticular diffusion model (Figure 17). According to Webber and Morris, the kinetic expression of intraparticle diffusion is given by the following equation (Equation (5)):

(5) $$q_{t}=k_{id}\cdot t^{\frac{1}{2}}+C$$

where *K_id_* is the intraparticle (pore) diffusion rate constant (mg/g·min^1/2^), and *C* (mg/g) is evaluated based on the regression line slope and intercept.

The computation of the primary linear plots area should not make junction with the origin, as the fluoride adsorption rate onto the modified zeolite was not exclusively conducted by pore diffusion. The increase of *K_id_* (Table 2), along with the concentration, suggests a predominant pore sorption process of fluoride ions onto modified zeolite.

### 2.9. Adsorption Isotherms

**Figure 18:**
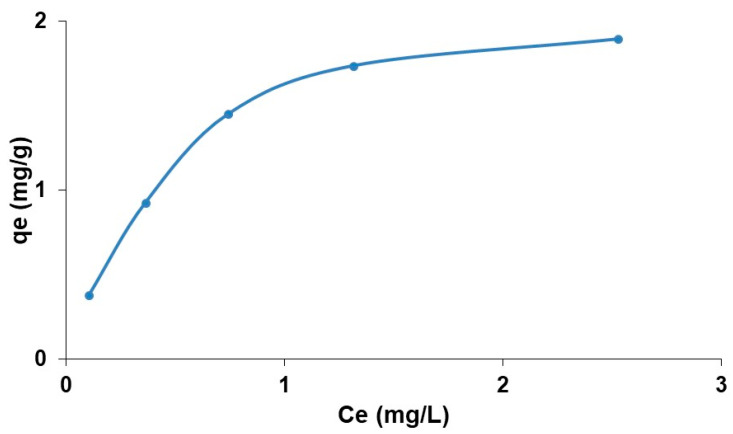
Fluoride adsorption isotherm on modified zeolite.

To study the fluoride adsorption isotherm equilibrium on modified zeolite, five NaF synthetic solutions were brought together, with a fluoride concentration ranging from 2 to 12 mg/L at an ambient temperature. Figure 18 plots the adsorbed quantities (q_e_) as a function of equilibrium fluoride concentrations (C_e_). It can be observed that the fluoride adsorption isotherm on modified zeolite is type I—the adsorbed capacity increases with an increase in the initial concentration until reaching a level corresponding to a diffusion balance between the solid and liquid phases. This isotherm is characteristic for molecular monolayers and is used to describe various ionic adsorptions.

**Figure 19:**
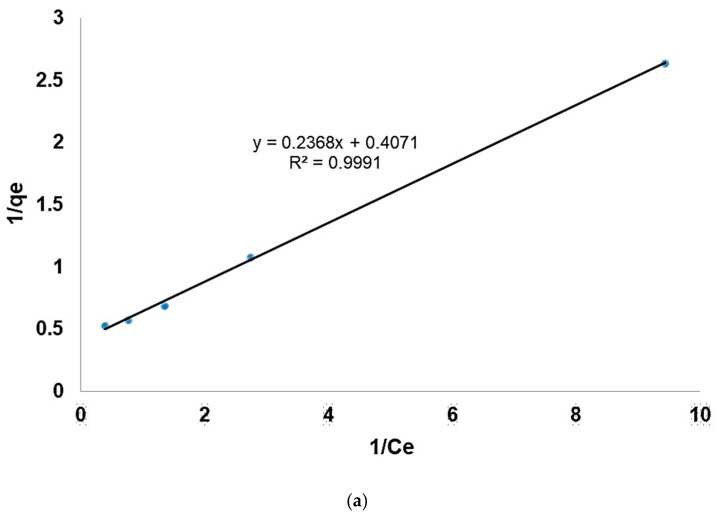

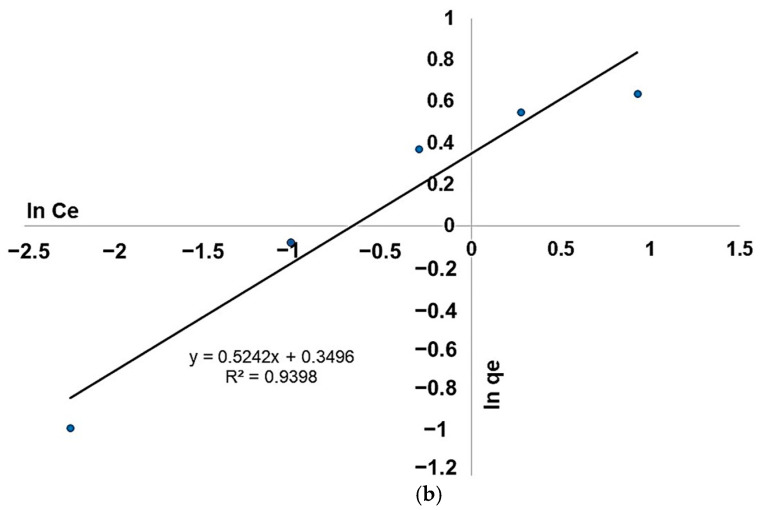
(**a**) Langmuir and (**b**) Freundlich models of the adsorption isotherms.

The adsorption isotherms were carried out by applying the Langmuir and Freundlich models. As presented in Figure 19a, the graphical representation of 1/q_e_ function of 1/C_e_ allows us to obtain the 1/K_q0_ slope and the origin ordinate 1/q_0_. The evaluation will provide the equilibrium factors (q_0_ and K) required for the Langmuir model investigation (Table 3). The linear Freundlich model presented in Figure 19b was obtained by plotting ln(q_e_) function of ln(C_e_), providing a line with 1/n slope and ln(K_f_) as y-axe intercept.

The correlation factor, as well as the other parameters, indicate that the Langmuir model is suitable to describe the fluoride adsorption process on the modified zeolite. A similar result was reported for fluoride removal using TiO_2_ and TiO_2_-SiO_2_ nanocomposites [52]. The results show that the adsorption site at the modified zeolite surface is energetically equivalent, and each site can bind a single molecule. Additionally, the RL value is less than 1; therefore, the experimental conditions are favorable for fluoride ion adsorption on substrates. The adsorption occurs as a single layer, and there is no interaction between the adsorbed molecules.

### 2.10. Thermodynamic Study

**Figure 20:**
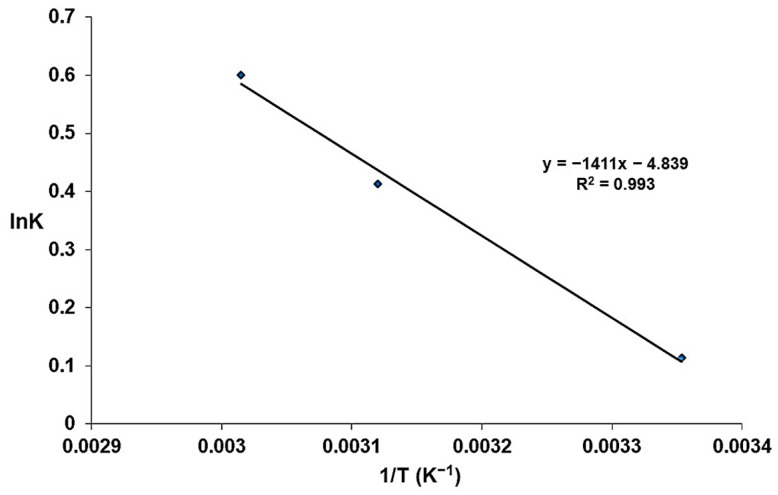
Representation of lnK as a function of temperature (1/T).

Thermodynamic parameters such as standard free enthalpy ΔG°, standard enthalpy ΔH°, and standard entropy ΔS° were determined using Equations (6)–(8) [53]:

(6) $$K=\frac{q_{e}}{C_{e}}$$

(7) $${\Delta G}^{{}^{\circ}}=\Delta H^{{}^{\circ}}-\;T\Delta S^{{}^{\circ}}$$

(8) $$\ln\;K={\frac{\Delta S}{R}}^{{}^{\circ}}-\frac{{\Delta H}^{{}^{\circ}}}{R}\;*\frac{1}{T}$$

where *K* is the distribution constant; *q_e_* represents the equilibrium adsorption capacity (mg/g); *C_e_* is the solute equilibrium concentration in solution (mg/L); R is the ideal gas constant (J/mol·K); and T represents the absolute temperature (K). Figure 20 shows the lnK plot vs. 1/T based on the above equations.

The thermodynamic parameters of the adsorption were evaluated at different temperatures, and the results are presented in Table 4.

The negative values of the free enthalpy ΔG° indicate that the adsorption of fluoride ions on the modified zeolite is a spontaneous process [17,54,55]. The decrease of ΔG° with increasing temperature is another parameter that indicates the adsorption takes place without additional outputs. On the other hand, the positive value of the enthalpy ∆H° shows that the adsorption process is endothermic, which confirms the kinetic and isotherm evaluations. Additionally, the entropy ∆S° exhibits positive values associated with a good affinity between the adsorbed ions and the modified zeolite [56].

## 3. Materials and Methods

### 3.1. Modified Zeolite

**Scheme 1:**
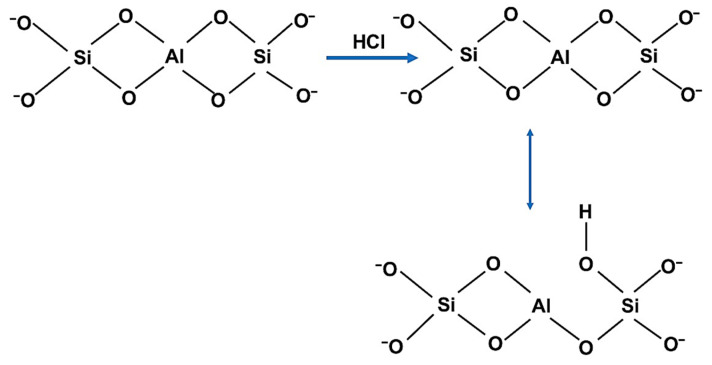
Clinoptilolite structural changes during HCl treatment.

Clinoptilolite ((Ca,Na)_2_Al_2_Si_7_O_18_·6H_2_O, Sigma-Aldrich, St. Louis, MO, USA) was the zeolite used in this study for fluoride adsorption. In order to increase the porosity, the zeolite was thermally treated at 600 °C for 2 h in a muffle furnace. After cooling, it was soaked in HCl (4%, Vwr chemicals Prolabo, Radnor, PA, USA) solution (4%) at 65 °C for one hour (Scheme 1).

**Scheme 2:**
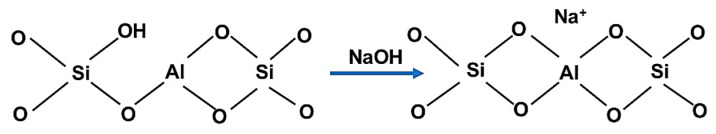
Structural changes during NaOH treatment.

In the second step, the material was pumped into a NaOH solution (1 mol/L, Novachim, Bucharest, Romania) for one hour at 40 °C (Scheme 2).

**Scheme 3:**
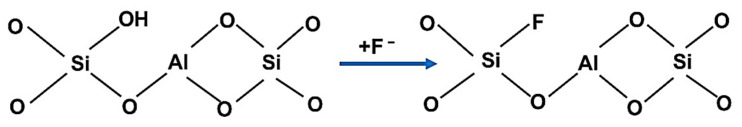
Ionic exchange in the zeolite material.

During the third step, the material was soaked in AlCl_3_ (10%, Sigma Aldrich) solution for 20 h (Equations (9)–(11) and Scheme 3). A comprehensive explanation of the mechanisms has already been presented in other papers [48,57,58].

(9) $${\mathrm{Al}}^{3+}\left(\mathrm{solution}\right)+3{\mathrm{Na}}^{+}\left(\mathrm{zeolite}\right)\to {\mathrm{Al}}^{3+}\left(\mathrm{zeolite}\right)+3{\mathrm{Na}}^{+}\left(\mathrm{solution}\right),$$

(10) $$\mathrm{Zeolite}-{\mathrm{AlOH}}_{2}^{+}+F^{-}\to \mathrm{Zeolite}-\mathrm{AlF}+H_{2}O\;\left(\mathrm{neutral}\;\mathrm{pH}\right),$$

(11) $$\mathrm{Zeolite}-\mathrm{AlOH}+F^{-}\to \mathrm{Zeolite}-\mathrm{AlF}+{\mathrm{OH}}^{-}\left(\mathrm{neutral}\;\mathrm{pH}\right),$$

Finally, the obtained mass was placed in an oven overnight at 100 °C, and the modified zeolite was used for fluoride ion adsorption studies.

#### 3.1.1. Feed Solution

The stock solution was prepared by dissolving 0.221 g of sodium fluoride (NaF) in one liter of distilled water. The volumetric flask used was made of polyvinyl chloride (PVC). The sodium fluoride was of analytical grade (Merck, Darmstadt, Germany). The working fluoride solution was prepared by diluting the stock solution with distilled water until the required concentration was reached. All the experiments were carried out in three phases: (i) the contact between the fluoride solution and the adsorbent, (ii) filtration, and (iii) fluoride ion concentration measurement.

#### 3.1.2. Fluoride Ions Analysis

The ionometric assay was performed using selective electrodes for fluoride ions. This principally consisted of measuring the membrane potential, representing the potential difference between the internal membrane surface, in contact with a reference solution, and the external membrane surface of the membrane. The difference was measured using two combined electrodes: (i) a reference electrode (Metrohm brand 6.0726.100 Ag/AgCl electrode) and (ii) a specific fluoride electrode (Metrohm brand 6.0502.150). In order to define the dose of the F^−^ ions, TISAB solution was prepared as follows: in a 1-L flask containing about 500 mL of distilled water, 57 mL of acetic acid (Sigma Aldrich) and 58g of NaCl (99%, Sigma Aldrich) were added. The solution pH was adjusted to 5.5 with sodium hydroxide (NaOH, 98%, Novachim) as a pellet. Distilled water was added up to the gauge line. In order to plot the calibration curve, standards of different NaF concentrations were prepared. The stock solution in NaF was 100 mg/L.

### 3.2. Structural and Composition Analysis of the Adsrobent

The FTIR analysis was made with a VERTEX 70 Fourier transform spectrometer (Bruker, Berlin, Germany) over a range from 400 to 4000 cm^−1^ with a resolution of 4 cm^−1^. Diffraction pattern was evaluated using a Discover 8 Difractometter (Bruker, Berlin, Germany). The diffraction scan was performed between relevant 2 theta angles (20–60°) with steps of 0.05 degree and a rate of 0.03 s/step. The active surface was evaluated with a porosimeter (Tristar II Plus Model, Micromeritics, Norcross, GA, USA) measuring N_2_ adsorption–desorption isotherm.

### 3.3. Batch Adsorption Studies and Influence of Adsorbent Mass

Prior to each adsorption test, the adsorbent had been previously dehydrated in an oven at 100 °C for one hour. This step served to remove water molecules, thus freeing up adsorption sites. In PVC bottles containing the solution that needed to be treated, different adsorbent quantities were added. The solution was stirred using a VELP brand multi-station stirrer for a well-determined time. After adsorption, the residue was filtered. Each experiment was conducted three times, and average values were reported.

The adsorption of fluoride ions on the modified zeolite was studied by varying the amount of adsorbent from 0.1 to 0.6 g for a fluoride concentration of 5 mg /L. The pH was adjusted to 6.7.

### 3.4. Influence of Contact Time

The period required to reach the adsorption equilibrium is necessary in order to identify the specific points needed to construct the isotherm, as well as its nature. Considering adsorption is a process based on the pollutant migration from the liquid phase to the solid phase, the transfer period between the two media represents a limiting factor.

Adsorption kinetics tests were carried out for different contact times (ranging from 0 to 180 min) in order to evaluate the period required to reach the pseudo-equilibrium state of fluoride ion binding on the adsorbent, using the modified zeolite.

The percentage evaluation of the fluoride ions adsorption on the modified zeolite was directly carried through the following relation Equation (12):

(12) $$\%Ads=\frac{\left({\left[F^{-}\right]}_{0}-{\left[F^{-}\right]}_{t}\right)}{{\left[F^{-}\right]}_{0}}*100,$$

The adsorption capacity was calculated based on Equation (13):

(13) $$q_{t}=\left({\left[F^{-}\right]}_{0}-{\left[F^{-}\right]}_{t}\right)*\frac{V}{m},$$

where [*F*^−^]_0_ represents the initial fluoride concentration (mg/L); [*F*^−^]_t_ constitutes the fluoride concentration at time *t* (mg/L); *V* expresses the solution volume; and m represents the adsorbent mass.

### 3.5. The pH and Temperature Influence

The pH values were adjusted by adding HCl (0.01 M, Vwr chemicals Prolabo) or NaOH (1 M, Novachim). In a series of bottles, a constant mass of 0.5 g of adsorbent was inserted; the fluoride aqueous solution (100 mL) with 5 ppm concentration and different pH was added.

In order to apprehend the thermodynamic phenomenon of fluoride adsorption on modified zeolite, the adsorption experiments were carried out by varying the solution temperature from 25 to 60 °C. The tests were performed using 100 mL solution volume with 5 mg/L concentration, pH 6.2, and adsorbent dosage of 0.5 g. Each experiment period was 180 min.

### 3.6. Effect of Co-Existing Ions

Drinking water contains several other ions, especially anions. These anions, similarly to fluorides, tend to be adsorbed on the zeolite, thus modifying the adsorption capacity of the material.

In order to evaluate the influence of other anions on the de-fluoridation rate, several tests were performed in the presence of SO_4_^2−^, Cl^−^, and HCO_3_^−^ at different concentrations (200, 300, 400, and 500 mg L^−1^) while maintaining the other fixed parameters.

## 4. Conclusions

The adsorption of fluoride ions on modified zeolite was evaluated, and the influence of the adsorbed amount, stirring, initial fluoride concentration, pH, temperature, and co-existing ions was evaluated. The results indicate that powder zeolite has a higher adsorption capacity (>90%) compared with granular zeolite (<60%). Increasing the adsorbent amount will increase the adsorption capacity until a certain point, wherein the equilibrium between the fluoride concentration and adsorbent is established. Using the dynamic working regime with 350 rpm will increase the adsorption efficiency from 93.7% to 98.5%. This study shows that most of the fluoride adsorption occurs in the first 50 min of the experiment, and the adsorption capacity increased from 0.93 to 1.74 mg/g when the initial fluoride ion concentration increased from 5 to 10 mg/L. Adsorption is also aided by acidic pH (2.7) and higher temperatures (58.7 °C).

This study indicates that the influence of the initial fluoride concentration on the adsorption kinetics follows the pseudo-second-order pattern, especially after the first 60 min of the reaction. Negative values of the free enthalpy ΔG° correspond to spontaneous processes. A positive value of enthalpy and entropy shows that the adsorption process is endothermic, and there is a good affinity between the fluoride ions and the adsorbent material. Langmuir’s model best expresses the type of adsorption considering the fluoride ions adsorbed in monolayers without fluoride–fluoride interactions, which increase the order of their surface distribution on the adsorbent.

## Figures and Tables

**Table 1:** Comparison of fluoride removal by different adsorbents.

Adsorbent Type	Adsorbent Dosage (g/L)	Adsorption Capacity (mg/g)	References
Acid-modified alumina	14	69.52	[21]
Natural zeolites from Ethiopia	60	0.47	[36]
Aluminum modified zeolite	3	2.37	[37]
Lanthanum hydroxide modified magnetites	100	1.42	[38]
Marble apatite-CM	10	4.23	[39]
Lanthanum oxyhydroxides anchored commercial granular activated carbon	3.33	9.98	[40]
Modified clinoptilolite	0.5	1.74	This work

**Table 2:** Kinetic parameters for pseudo-first-order, pseudo-second-order models, and intraparticle diffusion model.

Kinetic Parameters	Initial Concentrations of Fluoride (mg/L)		
	5	8	10
Pseudo-first-order model			
q_eexp_ (mg/g)	0.927	1.451	1.736
q_eth_ (mg/g)	0.108	0.326	0.384
K_1_ (min^−1^)	0.039	0.032	0.053
R^2^	0.947	0.959	0.947
Pseudo-second-order model			
q_eexp_ (mg/g)	0.927	1.451	1.736
q_eth_ (mg/g)	0.931	1.468	1.785
K_2_ (g/mg min)	1.516	0.376	0.337
R^2^	1	0.999	0.999
Intraparticle diffusion model			
K_id_	0.011	0.034	0.046
C	0.813	1.101	1.302
R^2^	0.704	0.803	0.687

**Table 3:** Langmuir and Freundlich parameters.

Langmuir Model				Freundlich Model		
q_0_ (mg/g)	K_L_ (L/mg)	R^2^	R_L_	K_f_	n	R^2^
2.456	1.706	0.999	0.124	1.418	1.907	0.939

**Table 4:** Thermodynamic parameters relating to the adsorption of fluoride on modified zeolite.

Thermodynamic Parameters	Temperature (°C)		
	25.2	47.5	58.7
ΔG ° (K·J·mol^−1^)	−0.266	−1.163	−1.613
ΔH ° (K·J·mol^−1^)	11.731	11.731	11.731
ΔS ° (J·mol^−1^·K^−1^)	40.231	40.231	40.231

## Data Availability

Data will be available upon request.
